# Augmented orexin/hypocretin signaling underlies negative affect during acute oxycodone abstinence in rats

**DOI:** 10.21203/rs.3.rs-7652324/v1

**Published:** 2025-10-13

**Authors:** Kimberly A. Newman, Victoria A. Stiritz, Jeff Cheng, Akihiro Yamanaka, Hiroyuki Mizoguchi, Morgan H. James, Gary Aston-Jones

**Affiliations:** 1 Brain Health Institute, Rutgers University and Rutgers Biomedical and Health Sciences, Piscataway, New Jersey.; 2 Rutgers Addiction Research Center, Brain Health Institute, Rutgers Health, Piscataway, NJ, 08854, USA; 3 School of Graduate Studies, Rutgers Health, Newark, NJ, USA.; 4 Neurobehavioral Research Laboratory, Research & Development, U.S. Department of Veterans Affairs, VA New Jersey Health Care System, East Orange, NJ, USA.; 5 Chinese Institute for Brain Research, Beijing (CIBR), Beijing, 102206, CHINA; 6 Department of Neuropsychopharmacology and Hospital Pharmacy, Nagoya University Graduate School of Medicine, Nagoya, JAPAN; 7 University of Sydney, Sydney, AUSTRALIA; 8 Department of Psychiatry, Robert Wood Johnson Medical School, Rutgers University, Piscataway, New Jersey, USA.

**Keywords:** Withdrawal, depression, anxiety, opioid use disorder, negative affect, hyperkatifeia

## Abstract

Opioid use disorder (OUD) is associated with a withdrawal-induced negative affective state, which contributes to uncontrolled use via negative reinforcement. The orexin (hypocretin) system is implicated in physical opioid withdrawal. We sought to test whether the orexin system is involved in negative affect during withdrawal from chronic prescription opioid exposure. Rats received non-contingent saline or oxycodone over 21d and were assessed for behaviors indicative of negative affect. We tested whether chronic oxycodone was associated with changes in orexin cell number or activity. We used chemogenetics in transgenic rats to modulate the activity of orexin neurons to determine their functional role in development of negative affect. Chronic oxycodone induced a negative affective state during acute abstinence that included relative weight loss, allodynia, and anhedonia-, anxiety, and despair-like behaviors. Oxycodone treatment was associated with an increase in the number and activity of orexin-immunoreactive neurons; the magnitude of negative affect severity directly correlated with the number of activated orexin neurons during acute abstinence. Chronic chemogenetic inhibition of orexin neurons concomitant with oxycodone exposure attenuated development of affective symptoms. Our data support a role for the orexin system in opioid withdrawal-associated negative affect and highlight this system as a potential treatment target for OUD.

## INTRODUCTION

Opioid use disorder (OUD) is a chronic and relapsing disorder that continues to be a public health emergency. Misuse of prescription opioids is a significant contributing factor to OUD^1,2^ with the widely prescribed analgesic oxycodone being one of the most commonly misused opioids^3^. Approximately 75% of heroin users seeking treatment reported first using prescription opioids, including oxycodone^3^, emphasizing the importance of prior prescription opioid exposure in the development of OUD.

Although the rewarding properties of opioids are important for initial drug use, aversive withdrawal states may contribute to the uncontrolled drug use that characterizes OUD^4–6^. The withdrawal state consists of both physical symptoms, including nausea, diarrhea, and hyperalgesia (increased pain sensitivity), and negative affective symptoms, most notably anxiety, anhedonia, and dysphoria (sometimes collectively referred to as hyperkatifeia^4–5^). Hyperalgesia (increased pain sensitivity) in particular has been shown to be exacerbated by a negative emotional state^4^. Relief from the withdrawal state via continued drug use may perpetuate ongoing drug use through negative reinforcement processes^4–8^.

Orexins A and B (also known as hypocretins 1 and 2) are neuropeptides exclusively produced by neurons in caudal hypothalamus that project broadly throughout the central nervous system and regulate a wide range of physiological processes by acting at two G-coupled receptors (orexin-1 receptors, Ox1Rs; orexin-2 receptors, Ox2Rs)^9,10^. Orexin cells play a critical role in drug-seeking; initial studies found that orexin neurons were Fos-activated in proportion to morphine conditioned place preference (CPP), and that stimulation of orexin neurons reinstated an extinguished morphine CPP^11^. Recent reports from our lab and others of increased numbers of orexin-expressing neurons following drug exposure^19–22^ have led to the hypothesis that a reserve population of orexin neurons may be recruited to drive addiction-like states^23^.

Multiple reports indicate that the orexin system plays a role in drug seeking for several opioids, including heroin, remifentanil, fentanyl, and oxycodone^12–15^. Additional studies have explored the roles of orexin signaling in physical withdrawal states. Physical symptoms of opioid withdrawal are attenuated in orexin knock-out mice^16^ and by Ox1R antagonism^17,18^, and pretreatment with an Ox1R antagonist reduces withdrawal-induced increases in Fos expression in several brain areas^17,18^. To date however, the role of orexin neurons in the negative affective state that accompanies physical withdrawal symptoms remains to be tested.

Here, we sought to examine the role of the orexin system in negative affective states associated with acute abstinence from the prescription opioid oxycodone. First, we established an animal model of non-contingent oxycodone exposure that promotes a multifaceted negative affective state during acute abstinence from oxycodone. We then used this model to test whether orexin neurons are important for the development of these outcomes. Our findings indicate that orexin signaling is involved in the development of negative affect during opioid abstinence, and that modulation of orexin signaling may offer a novel approach to prevent such adverse withdrawal states and therefore to attenuate OUD development.

## MATERIALS AND METHODS

### Animals.

**Figure 1.**
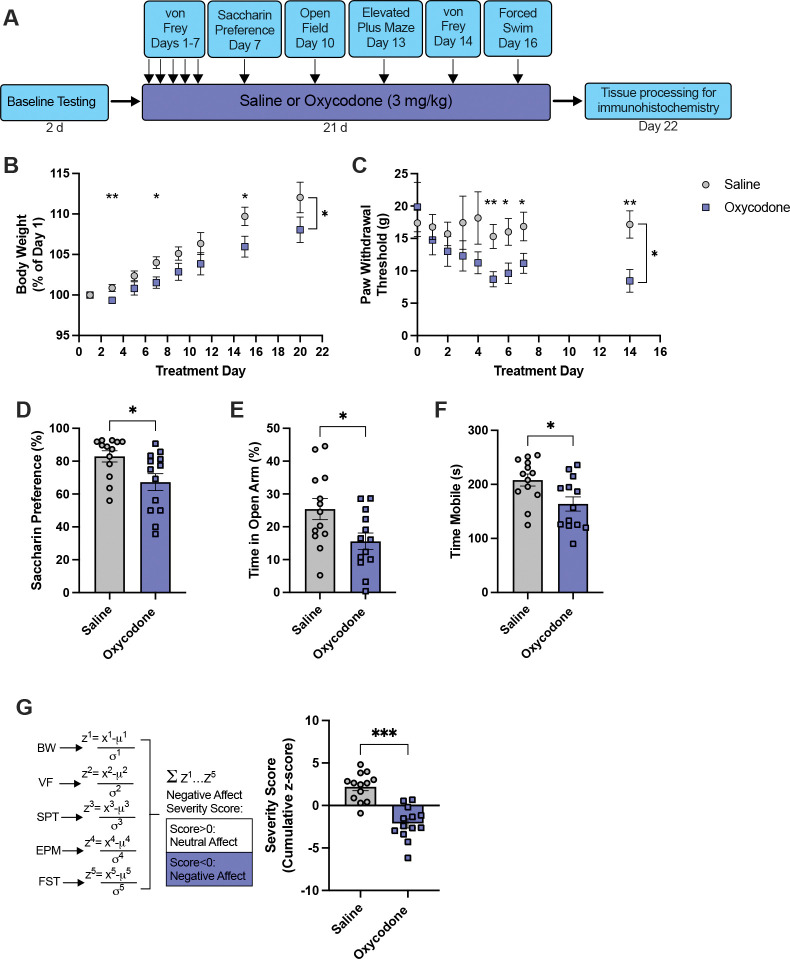
Chronic non-contingent oxycodone is associated with signs of negative affect during acute abstinence in male rats. **A)** Timeline of behavioral testing. Male rats were treated with saline (n=13) or oxycodone (n=13) twice daily for 21d and assessed for signs of negative affect during acute abstinence (16h after the last oxycodone injection). **B-C)** Compared to male saline controls, male oxycodone-treated rats exhibited reduced weight gain and lower paw withdrawal thresholds (allodynia) across the 21d of treatment (mixed-effects analyses with Holm-Sidak’s tests). **D)** Oxycodone-treated rats exhibited lower saccharin preference (increased anhedonia), **E)** increased anxiety-like behavior in the elevated plus maze, and **F)** increased passive coping behavior indicated by reduced mobility in the forced swim test (unpaired t-tests). **G)** Cumulative affect severity scores were generated for each individual by summing the z-scores from the five affective measures. Male oxycodone-treated rats showed greater negative affect (lower severity score) compared to saline controls (Kolmogorov-Smirnov test). BW, body weight; VF, von Frey; SPT, saccharin preference test; EPM, elevated plus maze; FST, forced swim test. *p<0.05, **p<0.01, ***p<0.001, all comparisons showing difference between saline and oxycodone.

Adult male Sprague Dawley (SD) rats were obtained from Charles River Laboratories (Kingston, NY). Male and female BAC transgenic orexin-cre rats on a Long Evans (LE) background were bred at Rutgers University from founders gifted by Akihiro Yamanaka (Nagoya University, Japan), described previously^24,25^. Male SDs were used in the initial experiment in Fig. 1; all subsequent experiments used LE rats from our in-house colony. Male and female rats weighed 494±70g and 248±38g, respectively, at the start of experiments. Rats were pair-housed under a 12:12h reverse light cycle (lights off at 0800h and on at 2000h) and given *ad libitum* access to food and water. All procedures were conducted in accordance with the NIH Guide for the Care and Use of Laboratory Animals and approved by Rutgers University Institutional Animal Care and Use Committee.

### Drugs.

Oxycodone HCl powder, clozapine-N oxide (CNO), and naloxone were obtained from the National Institute of Drug Abuse Drug Supply Program. Oxycodone HCl powder was dissolved in 0.9% sterile saline to a concentration of 3 mg/ml and injected at a volume of 1 ml/kg (i.p.). CNO was dissolved in 1% DMSO in sterile saline to a concentration of 1 mg/ml and given at a volume of 1ml/kg. Naloxone was prepared in sterile saline to a concentration of 0.5 mg/ml and injected at a volume of 1 ml/kg.

### Experiment 1a: Evaluating the effect of chronic oxycodone on negative affect behaviors in acute abstinence.

Fig. 1A illustrates the experimental timeline. Following at least 2d of habituation during which baseline measurements for the von Frey test (described below) and body weights were taken, male (n=26) and female (n=24) rats received twice daily injections of 0.9%. saline (1 ml/kg, i.p.) or oxycodone (3 mg/kg, i.p.) between 1000–1100h and 1600–1700h for 21d. Immediately prior to the 1000h injection (i.e., ~16h after the prior day’s injection and therefore during acute abstinence), behavioral signs of negative affect were assessed. Measures included weight gain, allodynia, and anxiety-, anhedonia-, and passive coping-like behaviors (described below). Behavioral tests progressed from least stressful (von Frey Test) to most stressful (Forced Swim Test).

#### von Frey Test (VF):

Mechanical hypersensitivity, an indicator of allodynia, was assessed in rats with the von Frey test using the up-down method, as previously described^14^. Briefly, von Frey filaments were applied to the mid-plantar surface of the rat’s hind paw for 2s and a positive response was scored for a retraction of the hind paw. Baseline paw withdrawal thresholds were determined by measuring paw withdrawal thresholds for 2 sessions prior to saline or oxycodone treatment. Paw withdrawal thresholds were assessed during the first week of treatment and again on Day 14.

#### Saccharin Preference (SPT):

On Day 4 of treatment, animals were temporarily switched to single-housing and habituated to two bottles, one containing tap water and the other containing 0.1% saccharin dissolved in tap water, both placed on the home cage for 2 consecutive days. Access to both bottles was given through sipper spouts and the left-right position of the bottles was alternated 24h later. After removal for 24h, the two bottles were reintroduced to the home cage on Day 7 for 2h, with the left-right position of the bottles alternated after 1h. Preference for saccharin was calculated using the formula: saccharin intake/(water intake+ saccharin intake) x100.

#### Open Field Test (OFT):

Animals were placed in novel locomotor chambers (clear acrylic, 42 cm × 42 cm × 30 cm) equipped with SuperFlex monitors (Omintech Electronics Inc., Columbus, OH) containing a 16 × 16 infrared light beam array for horizontal activity. Activity was recorded for 2h by Fusion SuperFlex software and reported as distance traveled (cm).

#### Elevated Plus Maze (EPM):

The elevated plus maze (Med Associates, St. Albans, VT) consisted of four arms (each 50 cm × 10 cm) and was elevated 50 cm above the floor. Rats were placed on the open arm facing the junction of the EPM and given 15 min to explore the maze. The time spent in each arm was automatically recorded by a computer monitoring beam crosses at the beginning of each arm.

##### Forced Swim Test (FST):

The forced swim test was performed in a cylindrical Plexiglas tank measuring 38.5 cm high × 30.5 cm diameter (Instech Laboratories, Plymouth Meeting, PA). The tank was filled with tepid water (25–27 °C), and rats were subjected to a 15 min initial swim exposure 24h prior to the test swim session. On test day, animals were given a 5 min swim test. A video camera was used to record each test and amount of time spent mobile (swimming, diving, or climbing) was quantified for each rat by an investigator blind to treatment group.

### Experiment 1b: Evaluating the effect of chronic oxycodone on orexin cell numbers and activity.

#### Tissue Preparation for Immunohistochemistry:

Tissue was collected from animals (n=15) in Experiment 1a to determine the effect of oxycodone on orexin cell numbers and activity. On Day 21 of treatment, rats were given a single saline or oxycodone injection at 1000–1100h. The following day (24h after the final injection), rats were deeply anesthetized with sodium pentobarbital and transcardially perfused with 0.9% sterile saline followed by 4% paraformaldehyde. Brains were collected and postfixed overnight in 4% paraformaldehyde. The next day, brains were transferred to a 20% sucrose-PBS azide solution and stored at 4°C until sectioning. Brains were sectioned at 40 μm using a cryostat and sections were stored in PBS-azide at 4°C. A separate group of rats given oxycodone treatment twice per day for 21d were sacrificed at 1 week (n=7) or 2 weeks (n=5) of abstinence and tissue was processed for immunohistochemistry as above.

#### Immunohistochemistry:

Hypothalamic sections from rats given saline or oxycodone were incubated in primary antibody directed against mouse orexin-A (1:500, Santa Cruz Biotechnology, catalog number sc-80263) or rabbit anti-melanin-concentrating hormone (MCH; 1:5000, Phoenix Pharm, catalog number H-070–47) and 5% normal donkey serum (NDS) in phosphate buffered saline (PBS) with Triton-X overnight at room temperature (RT). The following day, sections were rinsed in PBS and incubated in donkey anti-mouse or anti-rabbit secondary antibodies, respectively, conjugated to Alexa-Fluor 488 or 594, in PBST for 2h. All sections were rinsed in PBS, followed by phosphate buffer (PB), mounted onto glass slides, and cover slipped using Fluoroshield Mounting Medium with DAPI (Abcam, Waltham, MA). Orexin combined with ΔFosB immunoreactivity was assessed by incubating 40-μm-thick coronal sections in mouse anti-orexin-A (1:500) and rabbit anti-Δ-FosB (1:1000 Cell Signaling, catalog number 14695s) in 5% NDS in PBST overnight at RT. Secondary antibodies were a cocktail of Alexa-Fluor 488-conjugated donkey anti-mouse and Alexa-Fluor 594-conjugated donkey anti-rabbit in which tissue was incubated for 2h.

#### Imaging and Quantification:

Coronal images of the orexin cell field (2.5–3.3mm caudal to bregma) were taken using a Zeiss Axio Zoom V16 microscope and tiled photographs were compiled at 20x magnification using Zen 2 imaging software (Carl Zeiss Microscopy). Cell counts were taken from 3 non-adjacent sections in both hemispheres using ImageJ software (NIH) by an investigator blinded to the experimental condition. Medial hypothalamus (MH; comprised of the dorsal medial hypothalamus and perifornical area) and lateral hypothalamus (LH) orexin neurons were quantified separately due to evidence of functional differences between these populations in rat^11,17,20^. Medial and lateral hypothalamic subregions of the orexin cell field were divided by a vertical line made 100 μm lateral to the fornix, as done previously^20,21^. Cell counts per hemisphere were averaged across the three sections to obtain one value per subject.

### Experiment 2: Chronic inhibition of orexin neurons during oxycodone

Fig. 5A illustrates the experimental timeline. A chemogenetic approach was used to chronically inhibit orexin neurons during oxycodone treatment. Male orexin-cre rats (n=17) were anesthetized with 2% isoflurane and administered rimadyl (5 mg/kg, s.c.) for analgesia. Glass pipettes (20 μm tip) were used to microinject a DIO-AAV containing the inhibitory DREADD hM4Di (AAV9-hSyn-DIO-hM4D(Gi)-mCherry) or a non-DREADD control virus (AAV9-hSyn-DIO-mCherry; Addgene, Watertown, MA) bilaterally in the orexin cell field (1 μl/side). Stereotaxic coordinates for the orexin cell field were 2.6 mm posterior to bregma, 1.5 mm lateral, and −8.5 mm ventral to skull surface. Rats recovered for at least 3 weeks before initiating oxycodone treatment.

As in Experiment 1a, following baseline measurements rats were given 21d of twice daily oxycodone or saline. To chronically inhibit orexin neurons, hM4Di rats were given the DREADD agonist CNO (n=8; 1 mg/kg, i.p.) 30 min prior to each oxycodone treatment. An oxycodone-treated control group was composed of rats that received mCherry control virus and twice daily CNO + oxycodone (n=3) or hM4Di rats that received twice daily vehicle (no CNO) + oxycodone (n=6). A second control group consisted of wild-type (WT) drug-naïve littermates (n=10, no viral transduction) that were given twice daily CNO + saline. Some animals were given naloxone 90 min prior to sacrifice on Day 22; results in Experiment 3 indicated no effect of this acute manipulation on cell numbers. All affective measures were assessed at ~16h after the afternoon oxycodone injection, as above, and in the absence of CNO.

Rats were transcardially perfused following 21d of oxycodone treatment and hypothalamic sections were labeled for orexin using Alexa-Fluor 488-conjugated donkey anti-mouse secondary antibody as described above to verify transgene expression and quantify numbers of orexin neurons. Expression of the DIO-AAV (with or without hM4Di) was visualized via endogenous mCherry fluorescence. Animals in the hM4Di group not showing robust viral transduction were removed from the behavioral analysis.

### Experiment 3: Naloxone-precipitated withdrawal following chronic oxycodone.

To determine whether our regimen of chronic oxycodone exposure led to physical dependence, and to evaluate whether physical withdrawal involved orexin neuron activity, orexin-cre rats microinjected with mCherry (control, n=5) or hM4Di (n=6) virus were given 21d of oxycodone (no chronic hM4Di inhibition). One day later, rats were given CNO (1 mg/kg, i.p.) followed 30 min later by oxycodone (3 mg/kg, i.p.) and another 30 min later by naloxone (0.5 mg/kg, s.c.). Additional littermates (no viral transduction or chronic oxycodone) were used as non-dependent control groups, receiving a single CNO injection followed 30 min later by saline (n=9) or oxycodone (n=9), and then naloxone (0.5 mg/kg sc) after an additional 30 min. Withdrawal symptoms (burying, abdominal writhing, teeth chattering, head swoops, jumps, ptosis, and piloerection) were quantified for 30 min following naloxone injection. Measurements for each withdrawal behavior were normalized using min-max normalization and summed for each rat to generate a global physical withdrawal score.

### Data analyses.

Data are expressed in figures as mean values ± 1 standard error of the mean. Statistics were performed using GraphPad Prism for Mac (Version 10.2.2, GraphPad Software Inc., La Jolla, CA) with an α level of 0.05. Body weight data and paw withdrawal thresholds were analyzed using mixed-design ANOVAs. Independent samples t-tests were used to evaluate changes in saccharin preference, time spent in the open arms of the EPM, mobility in the FST, distance traveled in the OFT, orexin cell number and activity, and MCH cell number. A cumulative affect severity score was created by summing the z-scores for five affective behaviors: (z1) paw withdrawal threshold change from baseline, (z2) body weight change from Day 1, (z3) percent saccharin preference, (z4) percentage of time spent in the open arm of the EPM and (z5) time mobile in the FST. As lower z-scores for each test were indicative of increased negative affect, a lower severity score indicated a more severe global negative affect phenotype. A similar approach has been used to calculate addiction severity in an animal model of opioid addiction^26^. Kolmogorov-Smirnov tests were used to compare cumulative affect severity scores between saline- and oxycodone-treated rats. A two-way ANOVA was used to evaluate the effects of sex and oxycodone treatment on affect severity scores. One-way ANOVAs were used to evaluate the effect of abstinence duration on orexin cell number and activity, the effect of chronic inhibition of orexin neurons on negative affect behaviors, and the effect of orexin neuron inhibition on the expression of physical withdrawal behaviors. Spearman correlations were used to determine relationships between orexin cell number or activity in orexin neurons vs. cumulative affect severity. Holm-Sidak’s multiple comparisons tests were used as multiple comparison tests when appropriate. One outlier was removed from the analysis of MH ΔFosB-expressing orexin neurons (Grubb’s outlier test, p<0.05).

## RESULTS

### Experiment 1a: Negative affective behaviors following acute abstinence from chronic oxycodone.

We first sought to determine whether our regimen of non-contingent oxycodone injections induced a negative affective state. Male and female rats were treated with saline (males, n=13; females, n=11) or oxycodone (males, n=13; females, n=13) twice daily for 21d and assessed for signs of negative affect during acute abstinence (~16h after the previous saline/oxycodone treatment). Assays for negative affect included weight gain, allodynia, and anhedonia-, anxiety- and passive coping-like behaviors (Fig. 1A).

There was an effect of treatment group and day on weight gain in males (mixed-effects analyses, F_1,24_=4.387, p=0.0469 and F_1.400,31.80_=62.18, p<0.0001), as well as an interaction between these factors (F_7,159_=2.023, p=0.0553), such that oxycodone rats gained less weight over the 21d treatment regimen (Fig. 1B). There was an effect of oxycodone treatment on allodynia assessed with paw withdrawal thresholds in males (Fig. 1C, mixed-effects analysis, F=_1,24_=4.586, p=0.0426); although thresholds did not differ between groups at baseline, prior to treatment (Holm-Sidak’s tests, p=0.5786), oxycodone-treated males exhibited significantly lower thresholds by Day 5 of treatment and thereafter (Holm-Sidak’s tests, p<0.05). Oxycodone-treated males exhibited lower saccharin preference than saline control rats, an indicator of anhedonia (Fig. 1D, unpaired t-test, t_24_=2.538, p=0.0181). In the EPM, oxycodone males spent significantly less time in the open arm of the maze compared to saline controls (Fig. 1E, unpaired t-test, t_24_=2.406, p=0.0242), indicative of increased anxiety-like behavior. Oxycodone males showed decreased mobility in the forced swim test, indicative of passive coping behavior (Fig. 1F, unpaired t-test, t_24_=2.557, p=0.0173). We then compared cumulative affect severity scores across treatment groups. In males, oxycodone treatment was associated with a significant increase in negative affect compared to saline control rats (Fig. 1G, Kolmogorov-Smirnov test, D=0.7692, p=0.0009).

**Figure 2.**
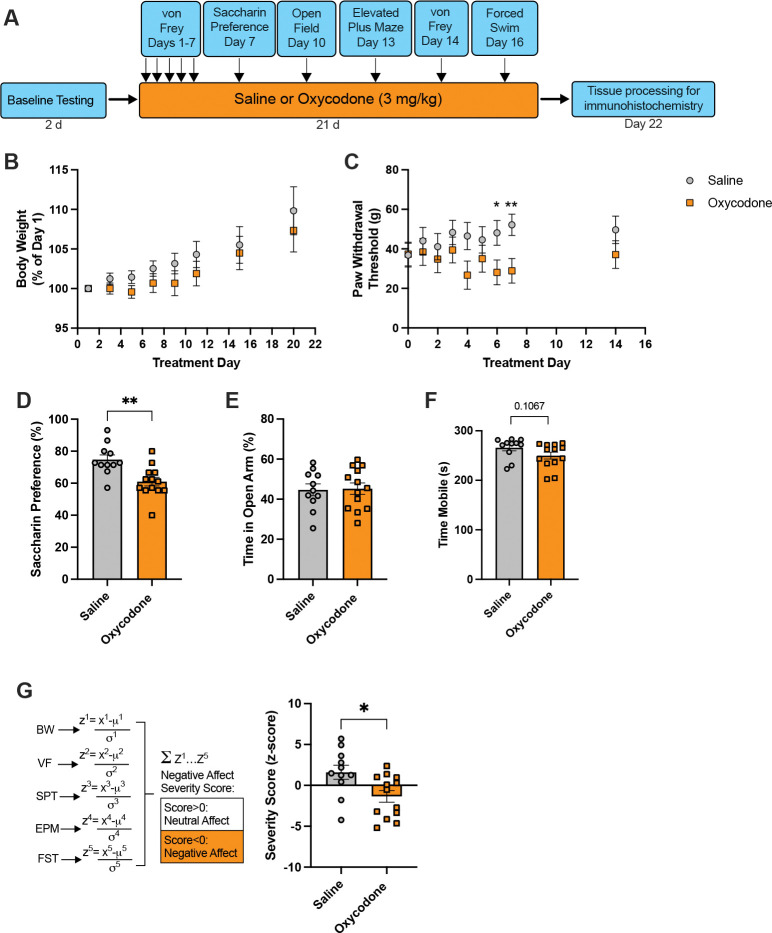
Chronic non-contingent oxycodone is associated with signs of negative affect during acute abstinence in female rats. **A)** Timeline of behavioral testing. Female rats were treated with saline (n=11) or oxycodone (n=13) twice daily for 21d and assessed for signs of negative affect during acute abstinence (16h after the last oxycodone injection). **B)** Compared to female saline control animals, female oxycodone-treated showed a trend towards less weight gain across the 21d of treatment (mixed-effects analysis). **C)** Oxycodone-treated females showed lower paw withdrawal thresholds (allodynia) at the end of the first week of treatment (mixed-effects analysis with Holm-Sidak’s tests). **D)** Oxycodone-treated females exhibited lower saccharin preference (anhedonia), **E)** no change in anxiety-like behavior in the elevated plus maze, and **F)** a trend towards reduced mobility (passive coping behavior) in the forced swim test (unpaired t-tests). **G)** Cumulative affect severity scores were generated for each individual by summing the z-scores from the five affective measures. Female oxycodone-treated rats showed greater negative affect (lower severity score) compared to saline controls (Kolmogorov-Smirnov test). BW, body weight; VF, von Frey; SPT, saccharin preference test; EPM, elevated plus maze; FST, forced swim test. *p<0.05, **p<0.01, all comparisons showing difference between saline and oxycodone.

Negative affect during acute abstinence from chronic oxycodone was also assessed in female rats (Fig. 2A). There was an effect of treatment day on weight gain in females (F_1.381,29.99_=15.75, p=0.0001), but a subtle decrease in weight gain in oxycodone-treated females compared to saline controls did not reach significance (Fig. 2B, mixed effects analysis, F_1,22_=0.8938, p=0.3547). There was a trend towards an effect of oxycodone on paw withdrawal thresholds (Fig. 2C, mixed effects analysis, F_1,22_=2.304, p=0.1433), with significantly lower paw withdrawal thresholds in oxycodone-treated rats at the end of Week 1 of treatment (Holm-Sidak’s tests, Day 6 vs. baseline, p=0.0346 and Day 7 vs. baseline, p=0.0097) but not at baseline (Holm-Sidak’s test, p=0.9511). Oxycodone-treated females exhibited lower saccharin preference, an indicator of anhedonia, than saline control rats (Fig. 2D, unpaired t-test, t_22_=3.482, p=0.0021). There was no effect of oxycodone on time spent in the open arm of the EPM in females (Fig. 2E, unpaired t-test, t_22_=0.1473, p=0.8842). Oxycodone-treated females showed decreased mobility in the forced swim test, although this trend did not reach significance (Fig. 2F, unpaired t-test, t_22_=1.682, p=0.1067). Cumulative affect severity scores were then compared across treatment groups. Oxycodone treatment in females was associated with a significant increase in negative affect compared to saline control rats (Fig. 2G, Kolmogorov-Smirnov test, D=0.5734, p=0.0397).

We also compared affect severity scores with both treatment and sex as factors. While there was an effect of treatment on affect severity score (two-way ANOVA, F_1,46_=31.59, p<0.0001), with both male and female oxycodone rats showing more severe negative affect than respective saline controls (Fisher’s LSD test, p<0.0001 and p=0.0028, respectively), there was no effect of sex on affect severity score (two-way ANOVA, F_1,46_=0.01562, p=0.9011). Thus, chronic oxycodone induced a similar degree of negative affect in male and female rats. Because severity scores were comparable between males and females, subsequent experiments utilized males only.

In a novel open field test, there was no significant difference between treatment groups in total distance traveled over the course of 1h in males (Supplemental Fig. 1A, unpaired t-test, t_16_=0.9252, p=0.3686) and females (Supplemental Fig. 1B, unpaired t-test, t_22_=1.696, p=0.1040). These results indicate that deficits in locomotor function were unlikely to have contributed significantly to the appearance of negative affect behaviors during acute abstinence in oxycodone-treated animals.

Together, these findings show that chronic non-contingent oxycodone is associated with the expression of a collection of negative affect behaviors in male and female rats during acute abstinence and thus serves as a model for the study of neural mechanisms underlying withdrawal-associated negative affect in OUD.

### Experiment 1b: Increased orexin cell number and activity in oxycodone-treated rats.

**Figure 3.**
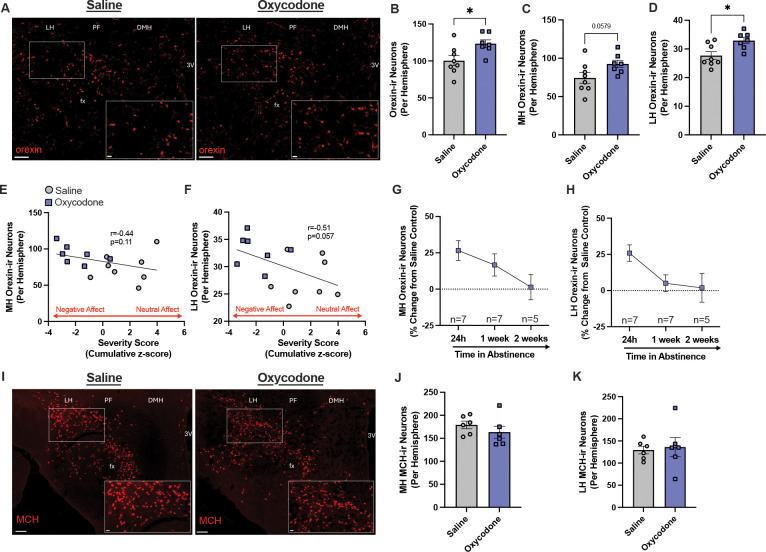
Orexin cell number is increased in oxycodone-treated rats and scales with negative affect severity. Orexin expression was assessed in saline- (n=8) and oxycodone-treated (n=7) rats sacrificed following 24h of abstinence. **A)** Low- and high-magnification (inset) images of orexin immunohistochemistry in sections of the hypothalamus of chronic saline- (left) or oxycodone- (right) treated animals sacrificed 24h after the final saline or oxycodone treatment. **B-C)** Compared to saline control animals, oxycodone-treated rats had more orexin-expressing neurons in both MH and LH (unpaired t-tests). **D-E)** In MH and LH, orexin cell numbers were negatively correlated with affect severity scores, such that rats with more orexin neurons exhibited stronger negative affect during treatment; in LH this correlation was significant (Spearman r correlations). **F-G)** Additional rats were given 21d of oxycodone and sacrificed following 1week (n=7) or 2 weeks (n=5) of abstinence. In MH and LH, the increase in orexin cell numbers with oxycodone at 24h of abstinence (expressed as percentage change from saline controls) decreased over 1 week or 2 weeks of abstinence, although not significantly (one-way ANOVAs with Holm-Sidak’s tests). **H)** Low- and high-magnification (inset) images of MCH immunohistochemistry in sections of the hypothalamus of a saline- (left) and oxycodone- (right) treated animal sacrificed 24h after the final saline or oxycodone treatment. **I-J)** The number of MCH-ir neurons in MH and LH did not differ between saline- (n=6) and oxycodone-treated animals (n=6; independent samples t-tests). Scale bar = 100μm in low magnification images and 20μm in high magnification images. LH, lateral hypothalamus; PF, perifornical area; DMH, dorsomedial hypothalamus; 3V, third ventricle; fx, fornix. *p<0.05.

A subset of male saline (n=8) and oxycodone (n=7) rats from Experiment 1a were assessed for orexin cell number and activity 24h after the final treatment (Fig. 3A). Affect severity scores in these rats were lower (greater negative affect) in oxycodone vs. saline rats (Kolmogorov-Smirnov, D=0.8571, p=0.0047).

Orexin cell numbers were increased in oxycodone-treated rats compared to saline controls (Fig. 3B, unpaired t-test, t_13_=2.627, p=0.0209). Orexin cell numbers were also quantified separately for lateral hypothalamus (LH) and medial hypothalamus (MH), as evidence points to functional differences between these neuronal subpopulations^11,17,20^. Orexin cell numbers were increased in both MH and LH in oxycodone-treated rats compared to saline controls (Fig. 3C–D, unpaired t-tests, t_13_=2.080, p=0.0579 and t_13_=2.900, p=0.0124, respectively).

We then sought to determine whether there was a relationship between individual orexin cell number and affect severity experienced during treatment. There was a strong trend towards a negative correlation between orexin cell number and affect severity score (Spearman r correlation, r=−0.4821, p=0.0711). In both MH and LH, there were trends towards negative correlations between orexin cell numbers and affect severity scores, such that individuals with more severe negative affect had more orexin neurons (Fig. 3E–F, Spearman r correlations, r=−0.4357, p=0.1063 and r=−0.5058, p=0.0565, respectively).

To determine whether observed increases in orexin cell numbers were sustained with longer abstinence, additional rats were given 21d of oxycodone and were sacrificed following 1 week (n=7) or 2 weeks of abstinence (n=5). In MH, there was no significant effect of abstinence time on orexin cell numbers (Fig. 3G, one-way ANOVA, F_2,16_=2.517, p=0.1121); there was no significant decrease in orexin cell numbers at 1 week of abstinence, but a trend towards decreased orexin cell numbers at 2 weeks of abstinence (Holm-Sidak’s tests, p=0.3472 and p=0.0773, respectively). In LH, there was an effect of abstinence time on orexin cell numbers (Fig. 3H, one-way ANOVA, F_2,16_=3.687, p=0.0482), with a trend towards decreased orexin cell numbers at 1 week and 2 weeks of abstinence (Holm-Sidak’s tests, p=0.0601 and p=0.0601, respectively).

Next, we determined whether changes in cell numbers in oxycodone-treated rats extended to other cell groups. MCH neurons are interdigitated with, but distinct from, orexin neurons in the hypothalamus^27,28^ (Fig. 3I). The number of MCH-ir neurons did not differ between saline and oxycodone-treated rats in MH or LH (Fig. 3J–K, independent samples t-tests, t_10_=1.03, p=0.3251 t_10_=0.287, p=0.7799, respectively). Thus, changes in cell numbers in oxycodone-treated rats are at least somewhat selective for orexin neurons.

**Figure 4.**
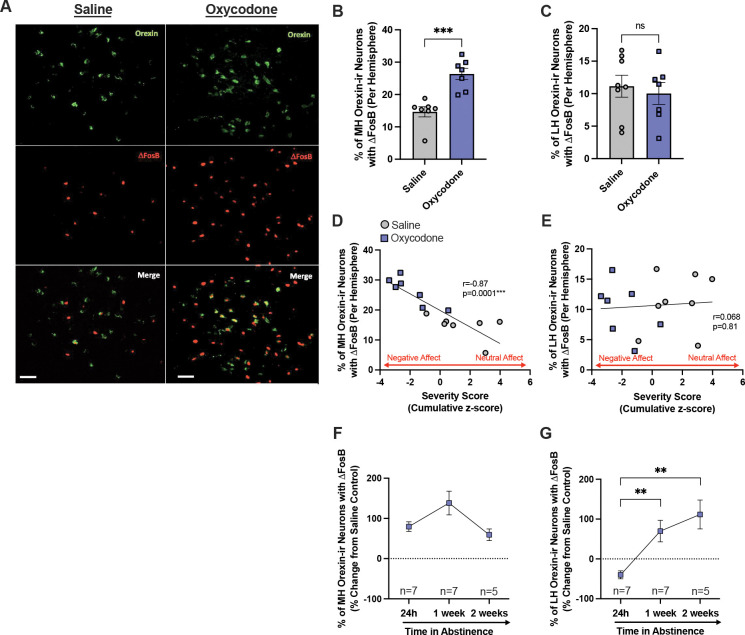
Orexin neuron activity is increased following chronic oxycodone and in MH scales with negative affect severity. Orexin activity (assessed with ΔFosB) was assessed in saline- (n=8) and oxycodone- (n=7) treated rats following 24h of abstinence. **A)** Immunohistochemistry of hypothalamic sections showing orexin (green) and ΔFosB (red) from a rat given chronic saline (left) or oxycodone (right). Scale bar = 50μm. **B-C)** Oxycodone-treated rats had a greater percentage of MH, but not LH, orexin neurons that expressed ΔFosB compared to saline controls (independent samples t-tests). **D-E)** In MH, affect severity scores were strongly correlated with percentages of ΔFosB-expressing orexin neurons, such that individuals with more severe negative affect had more activated orexin neurons; in LH there was no relationship (Spearman r correlations). **F-G)** Additional rats were given 21d of oxycodone and sacrificed following 1 week (n=7) or 2 weeks (n=5) of abstinence. In MH, the increase in ΔFosB-activated orexin cell numbers at 24h of abstinence (expressed as percentage change from saline controls) was sustained at 1 week and 2 weeks of abstinence. In LH, activation of orexin neurons with ΔFosB was significantly increased from 24h by 1 week and 2 weeks of abstinence (one-way ANOVAs with Holm-Sidak’s test). Panels B-C: ***p<0.001, ns=not significant, comparisons showing difference between saline and oxycodone. Panels D-E: ***p<0.001. Panels F-G: **p<0.01, comparisons showing 1 week and 2 weeks of abstinence vs. 24h of abstinence.

Previous work has shown that orexin neurons are Fos-activated during naloxone-precipitated opioid withdrawal^16–18^. Here, activity of orexin neurons was assessed in our model of repeated opioid abstinence using ΔFosB, a long-lasting transcriptional regulator proposed to play a role in drug-induced neural plasticity^29^ (Fig. 4A). Compared to saline control animals, oxycodone-treated rats exhibited a greater percentage of MH, but not LH, orexin neurons that expressed ΔFosB at 24h of abstinence (Fig. 4B–C, unpaired t-tests, t_12_=4.931, p=0.0003 and t_13_=0.4644, p=0.6501, respectively).

There was a strong relationship between numbers of MH ΔFosB-expressing orexin neurons and affect severity scores, such that rats with more activated MH orexin neurons had more severe negative affect (Fig. 4D, Spearman r correlation, r=−0.8725, p=0.0001). In LH, however, there was no correlation between numbers of ΔFosB-expressing orexin neurons and affect severity scores (Fig. 4E, Spearman r correlation, r=0.06786, p=0.8124).

We then determined whether orexin neuron activation levels as seen with ΔFosB changed over the course of abstinence. In MH, there was a strong trend towards an effect of abstinence time on numbers of ΔFosB-expressing orexin neurons (Fig. 4F, one-way ANOVA, F_2,16_=3.585, p=0.0517), with a trend towards an increase in ΔFosB activation of orexin neurons at 1 week of abstinence and no change from 24h at 2 weeks of abstinence (Holm-Sidak’s tests, p=0.1173 and p=0.5300, respectively).

In LH, there was an effect of time on percentage of ΔFosB-expressing orexin neurons (Fig. 4G, one-way ANOVA, F_2,16_=9.875, p=0.0016), such that ΔFosB activation of orexin neurons was elevated, above saline controls, at 1 week and 2 weeks of abstinence (Holm-Sidak’s tests, p=0.0046 and p=0.0015, respectively).

Together, these results demonstrate that chronic oxycodone is associated with an increase in orexin cell number and activity. Numbers of activated MH orexin neurons strongly predicted affect severity, indicating that MH orexin neurons in particular may be important for chronic oxycodone-associated negative affect.

### Experiment 2a: Attenuated development of negative affective behaviors following inhibition of orexin neurons during chronic oxycodone administration.

**Figure 5.**
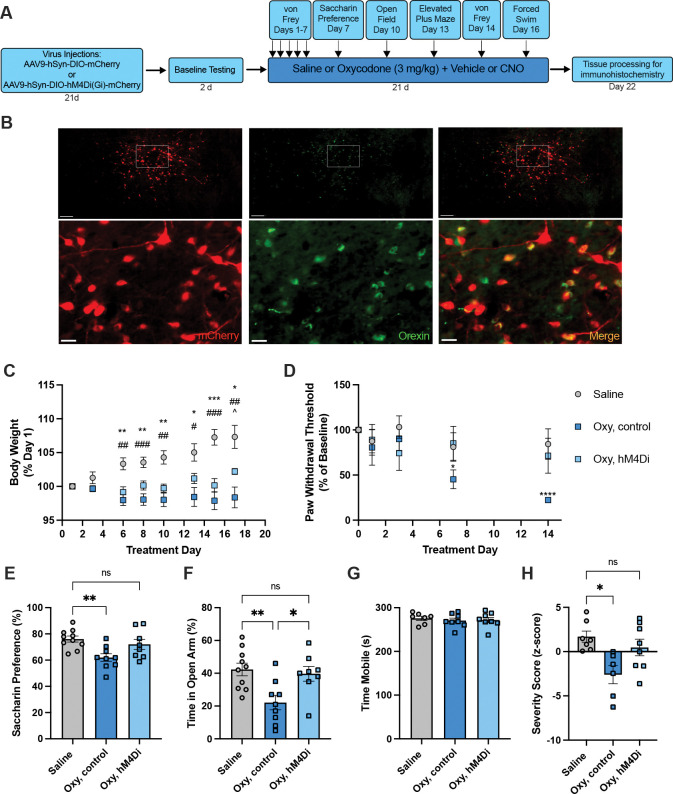
Chronic inhibition of orexin neurons prevents the development of negative affect associated with acute abstinence from oxycodone. **A)** Illustration of the experimental timeline (for details see Materials and Methods). During acute abstinence (16h following the last injection), saline control (n=10), oxycodone control (n=9), and oxycodone hM4Di (n=8; receiving daily hM4Di inhibition of orexin neurons) rats were tested on negative affect measures, in the absence of CNO. One day later, rats were sacrificed and tissue was processed for orexin immunohistochemistry. **B)** Low- (top) and high-magnification inset (bottom) images of expression of the AAV-DIO (seen with endogenous mCherry expression; left), orexin immunohistochemistry (middle), and merge (right) in the hypothalamus of a rat that received the DIO-AAV containing hM4Di. Scale bar = 100μm in low-magnification images and 20μm in high-magnification images. **C-D)** In oxycodone-treated hM4Di rats, CNO prior to each oxycodone treatment (to inhibit orexin neurons) partially attenuated decreased weight gain and attenuated the reduction in paw withdrawal thresholds (attenuated allodynia) seen with oxycodone treatment (mixed-effects analyses with Holm-Sidak’s tests). Inhibition of orexin neurons in hM4Di rats also attenuated oxycodone-associated decreases in **E)** saccharin preference and **F)** anxiety-like behavior in the EPM, but **G)** had no effect on mobility in the FST. **H)** Affect severity scores were attenuated in hM4Di rats given CNO (one-way ANOVAs with Holm-Sidak’s tests). Panel C: *p<0.05, **p<0.01, ***p<0.001, difference between saline and oxycodone hM4di; #p<0.05, ##p<0.01, ###p<0.001, difference between saline and oxycodone control; ^p<0.05, difference between oxycodone control and oxycodone hM4Di. Panel D: *p<0.05, Treatment Day 7 vs. baseline in oxycodone controls; ****p<0.0001, Treatment Day 14 vs. baseline in oxycodone controls. Panels E-H: *p<0.05, **p<0.01, ns=not significant, comparisons showing difference between treatment groups.

We next tested whether chronic inhibition of orexin neuronal activity could prevent the development of negative affect. Fig. 5A illustrates the experimental timeline. Transgenic orexin-cre rats (n=8) were injected into the hypothalamus with a DIO-AAV that expressed the inhibitory DREADD hM4Di. Three weeks later, twice daily oxycodone treatment for 21d was initiated. Thirty minutes prior to each oxycodone injection, animals received a systemic injection of the DREADD agonist CNO (1 mg/kg, i.p.) to inhibit orexin neurons. An orexin-cre oxycodone control group (n=9, no hM4Di activation) and a WT saline control group (n=10, no viral transduction or oxycodone) received twice-daily injections. All affective measures were assessed during acute abstinence (16h after the last saline or oxycodone injection, as above) and in the absence of CNO.

One day following treatment, rats were sacrificed and specificity of hM4Di expression was determined using dual label immunohistochemistry (Fig. 5B). Delivery of control or hM4Di-expressing DIO-AAV (expressing mCherry) into hypothalamus of orexin-cre rats resulted in robust transduction of orexin neurons surrounding the injection site, with 76.4% of orexin neurons expressing mCherry (n=13 hemispheres from 5 rats). Transgene expression was highly specific, with 88.6% of mCherry neurons expressing orexin.

There were effects of treatment day and group on body weight change (Fig. 5C, mixed-effects analysis, F_2.040,44.59_=5.655, p=0.0062 and F_2,24_=17.08, p<0.0001, respectively), and an interaction between treatment day and group (F_14,153_=6.981, p<0.0001). Both oxycodone control and hM4Di rats gained less weight than saline controls over the course of treatment (Holm-Sidak’s tests, p<0.05). However, oxycodone-associated deficits in weight gain were partially attenuated with hM4Di inhibition of orexin neurons. There was an effect of treatment day on change in paw withdrawal threshold from baseline in the von Frey test (Fig. 5D, mixed-effects analysis, F_3.287,64.92_=5.467, p=0.0015) as well as an interaction of treatment day with group (F_8,79_=2.132, p=0.0422); although oxycodone controls showed decreased paw withdrawal thresholds from baseline over the course of treatment, hM4Di rats did not, in line with saline controls (Holm-Sidak’s tests, Day 7 vs. baseline and Day 14 vs. baseline, p<0.05 for oxycodone controls and p>0.05 for hM4Di and saline controls). There was an effect of group on saccharin preference (Fig. 5E, one-way ANOVA, F_2,24_=6.110, p=0.0072), such that compared to saline controls, oxycodone controls had lower saccharin preference, but hM4Di rats did not (Holm-Sidak’s tests, p=0.0066 and p=0.3479, respectively). Similarly, there was an effect of group on time spent in the open arm of the maze in the EPM (Fig. 5F, one-way ANOVA, F_2,24_=6.841, p=0.0045), such that oxycodone control rats spent less time in the open arms of the maze compared to saline controls (Holm-Sidak’s test, p=0.0060), an effect that was reversed by hM4Di inhibition of orexin neurons (Holm-Sidak’s test, saline control vs. hM4Di, p=0.6638 and oxycodone control vs. hM4Di, p=0.0177). In the FST, there was no effect of group (Fig. 5G, one-way ANOVA, F_2,20_=0.2043, p=0.8169), with no effect of hM4Di inhibition of orexin neurons on swim mobility.

Affect severity scores were calculated as in Experiment 1a. There was a group effect on this cumulative measure (Fig. 5H, Krushkal-Wallis test, H=8.396, p=0.0095), such that oxycodone control rats had significantly greater negative affect than saline control animals (lower severity score; Dunn’s test, p=0.0118), an effect that was prevented by hM4Di inhibition of orexin neurons (saline control vs. oxycodone hM4Di, p=0.7154).

### Experiment 2b: Reduced numbers of orexin-expressing neurons following chemogenetic inhibition of orexin neurons during chronic oxycodone.

To determine the effect of inhibition of orexin neurons during chronic oxycodone administration on orexin cell numbers, the number of orexin-ir neurons was quantified in oxycodone control (mCherry; n=5) and hM4Di (n=5) rats that had received 21d of oxycodone treatment with CNO. At 24h after the final oxycodone treatment, there was a trend towards fewer orexin cells in hM4Di compared to control rats in MH (Supplemental Fig. 2A, unpaired t-test, t_8_=1.297, p=0.2307); in LH this decrease was significant (Supplemental Fig. 2B, unpaired t-test, t_8_=2.282, p=0.0519). Taken together, these results indicate that decreased orexin neural activity during oxycodone exposure prevents the development of negative affect behaviors and the associated increase in orexin-ir cell numbers in LH.

### Experiment 3: Attenuated expression of physical withdrawal following chemogenetic inhibition of orexin neurons during chronic oxycodone.

Previous studies found that orexin signaling is associated with physical symptoms of naloxone-precipitated withdrawal in morphine-dependent rats^16–18^. Here, we determined whether our regimen of oxycodone was associated with physical dependence, and whether orexin neurons are involved in the expression of physical withdrawal. Separate groups of hM4Di (n=6; Chronic Oxycodone hM4Di group in Supplemental Fig. 4) or oxycodone control (n=5; Chronic Oxycodone mCherry group) rats were given 21d of oxycodone treatment (no daily hM4Di inhibition). One day later, all rats received CNO 30 min prior to an oxycodone injection, which was followed 30 min later by naloxone (to induce an acute withdrawal response^30^). Two additional groups of drug-naïve rats (no viral transduction or oxycodone) were given CNO followed 30 min by a single saline injection (n=9; Saline group in Supplemental Fig. 4) or oxycodone injection (n=9; Acute Oxycodone group) and naloxone another 30 min later. Following naloxone, rats were sacrificed and specificity of hM4Di expression was determined using dual label immunohistochemistry.

Delivery of the DIO-AAV that expressed mCherry into the orexin cell field resulted in robust and specific transduction of orexin neurons, with 70.9% of orexin neurons expressing mCherry and 89.1% of mCherry neurons expressing orexin (n=14 hemispheres from n=7 rats).

There was an effect of treatment group on global withdrawal score (Supplemental Fig. 3, one-way ANOVA, F_3,25_=47.21, p<0.0001). Naloxone given to naïve rats after a single oxycodone injection produced a significantly higher global physical withdrawal score than when given after saline (Holm-Sidak’s test, p=0.0033). Withdrawal scores were significantly higher in rats that received 21d of oxycodone compared to a single oxycodone injection (Holm-Sidak’s test, Acute Oxycodone vs. Chronic Oxycodone mCherry, p<0.0001). This confirms that our chronic oxycodone regimen induces dependence by potentiating induction of a physical withdrawal response. These elevated withdrawal scores were attenuated with hM4Di inhibition of orexin neurons prior to naloxone (Holm-Sidak’s test, Chronic Oxycodone mCherry vs. Chronic Oxycodone hM4Di, p=0.0033), indicating that inhibition of orexin neurons prevented the induction of physical dependence.

Orexin cell numbers in mCherry rats given 21d of oxycodone followed by naloxone 30 min prior to sacrifice (n=4) did not differ from orexin cell numbers in oxycodone-treated rats in Experiment 1a that received no naloxone (n=7; unpaired t-test, t_9_=0.5058, p=0.6251), indicating that acute treatment of naloxone did not change numbers of orexin cells.

## Discussion

We describe a novel role for the orexin system in negative affect associated with acute abstinence from oxycodone. We show that chronic non-contingent oxycodone exposure induces signs of negative affect during abstinence which are associated with augmentation of orexin cell number and activity. Inhibition of orexin neurons during oxycodone treatment prevented the development of negative affect behaviors and dependence, indicating that these changes require chronic engagement of the orexin system. Together, these data indicate that dependence and withdrawal symptomology associated with oxycodone use might be prevented by concomitant treatment with therapeutics that attenuate orexin signaling.

Exposure to prescription opioids is a major contributing factor to the current opioid crisis^1–3^; however, the mechanisms underlying opioid dependence are unclear. Evidence supports the idea that addiction consists of three distinct stages including binge/intoxication, withdrawal/negative affect, and preoccupation/anticipation^4^. Negative affect serves as a critical source of motivation that helps drive compulsive drug-taking and thus represents a key step by which prescribed opioid use transitions to abuse. Indeed, data from clinical populations show that symptoms of negative affect are predominant during early abstinence and contribute to relapse^31,32^. Although studies of withdrawal-induced negative affect in animals are limited, recent studies reported elevated anhedonia-, anxiety-, and despair-like behaviors during protracted morphine abstinence in mice^33,34^. Here, we show that, in addition to producing hallmark signs of physical dependence (as previously shown with morphine^16–18^), oxycodone treatment was associated with several behaviors indicative of negative affect in rats during acute abstinence, including allodynia, anhedonia- and anxiety-like behavior, and passive coping, and therefore provides a useful preclinical model for these phenomena.

Prior research implicates the orexin system in motivated drug seeking. However, the role of orexin peptides in negative reinforcement produced by opioid dependence has been relatively unexplored; one study found that blockade of Ox2Rs in the paraventricular nucleus of the thalamus in rats attenuated conditioned place aversion to morphine withdrawal^35^, which is thought to reflect negative affect. Here, we found that oxycodone treatment was associated with an increase in orexin cell numbers but not in neighboring MCH neurons. Our results are consistent with prior studies showing that chronic exposure to drugs of abuse increases orexin expression^19–22^, leading to the hypothesis for ‘reserve’ orexin neurons in addiction^23^. These reserve neurons were previously linked with increased motivation for reward; data here indicate that recruitment of these cells might also be involved in negative affective states during withdrawal. This hypothesis is underlined by our observation that number of orexin neurons predicted negative affect severity. We speculate that orexin neurons may contribute to increased drug motivation via negative reinforcement, consistent with reports of correlations between drug seeking and numbers of Fos-activated orexin neurons^11,36^.

We also assessed the activity of orexin neurons using ΔFosB, a persistent transcription factor associated with neural activity and plasticity in addiction^29^. Prior studies found that ΔFosB accumulates in other brain neurons with repeated drug exposure^37–40^, and such expression was maintained over prolonged periods of withdrawal (~5 weeks)^41–42^. We found that ΔFosB in MH orexin neurons is upregulated within 24h after chronic oxycodone treatment. In contrast, upregulation of ΔFosB is delayed in LH orexin neurons and only present after longer withdrawal (1–2 weeks). Although not tested directly here, this may indicate that MH orexin neurons play a direct role in withdrawal-associated negative affect; this is supported by the fact that we saw a strong correlation between negative affect severity and number of ΔFosB-expressing orexin neurons in MH, but not LH. This is also consistent with the finding that opioid withdrawal precipitated by naloxone activates orexin neurons in MH, but not LH^17^. LH orexin neurons may be more prominently involved in increased motivation associated with withdrawal, as LH orexin neurons have been shown to regulate motivation for other drugs^20,43,44^. Indeed, overexpression of ΔFosB in other brain neurons promotes drug-seeking^45^, a critical function of LH orexin neurons^11,20,43,44,46,47^. Together, these results support a role for MH orexin neurons in negative affect associated with acute abstinence, and for LH orexin cells in enhanced drug-seeking during prolonged abstinence^48^.

We found that inhibition of orexin neurons prior to naloxone-precipitated withdrawal reduced the expression of physical withdrawal, consistent with prior reports^17,18^. Moreover, our results show that orexin neuron inhibition during oxycodone treatment prevented development of a negative affective phenotype associated with acute abstinence. Additionally, chronic inhibition of orexin neurons during oxycodone administration prevented development of increased orexin cell numbers in LH following oxycodone treatment. A potential mechanism of orexin-dependent orexin system plasticity could involve direct or indirect feedback from orexin neurons themselves or from downstream circuits; future experiments are needed to further illuminate these processes.

It is difficult to determine whether chemogenetic inhibition of orexin neurons prevented the development of negative affect via an orexin-dependent mechanism, as orexin neurons co-release other transmitters^49–52^, including dynorphin, which has been proposed to play a role in negative affective states during withdrawal^53^. Indeed, preprodynorphin mRNA is elevated in other brain regions following chronic drug exposure^54–59^, and dynorphin/kappa opioid receptor signaling contributes to physical and affective symptoms of drug withdrawal^57,59–62^. Further studies are warranted to discern the roles of orexin and dynorphin in the development and expression of oxycodone-induced negative affect.

In sum, we described a novel role for the orexin system in the development of negative affect associated with acute abstinence from oxycodone. We show that chronic oxycodone is associated with an increase in numbers and activity of orexin neurons and that development of these behaviors can be prevented by inhibition of orexin neuron activity. Together, our findings indicate that orexin neurons may play an important role in the negative reinforcement processes that underlie persistent opioid use in OUD. As a consequence, orexin-based medications offer multifaceted potential therapeutic benefits for the management of OUD.

## Supplementary Material

Supplement 1

## Data Availability

All data supporting the findings of this study are available within the paper and its Supplemental Data.
