# Supplementary material for: Augmented orexin/hypocretin signaling underlies negative affect during acute oxycodone abstinence in rats
Source: Res Sq. 2025 Oct 13:rs.3.rs-7652324. Preprint. [Version 1] doi: 10.21203/rs.3.rs-7652324/v1 (PMC12633195; doi:10.21203/rs.3.rs-7652324/v1)
Supplement: Supplement 1 [file NIHPPrs7652324v1-supplement-1.pdf]

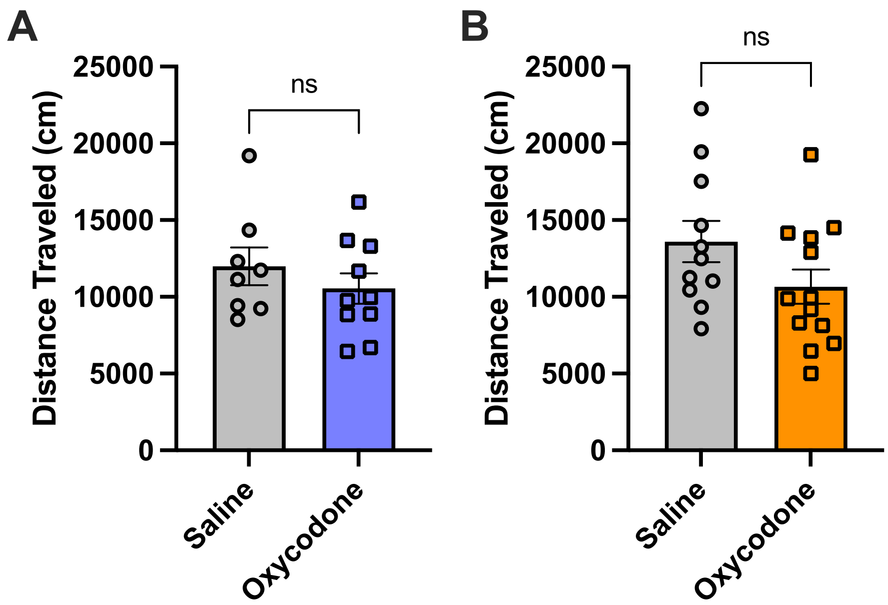

Supplemental Figure 1. Effects of acute abstinence from chronic oxycodone on locomotor activity in a novel open field test. On Day 10 of saline or oxycodone treatment, rats were tested on a novel open field test for 1h during acute abstinence (16h after the preceding oxycodone injection). **A)** There was no difference in distance traveled in the open field between male saline controls (n=8) and male oxycodone-treated rats (n=10). **B)** Similarly, there was no significant difference in distance traveled between female saline controls (n=13) and female oxycodone-treated rats (n=13). Unpaired t-tests, ns=not significant.

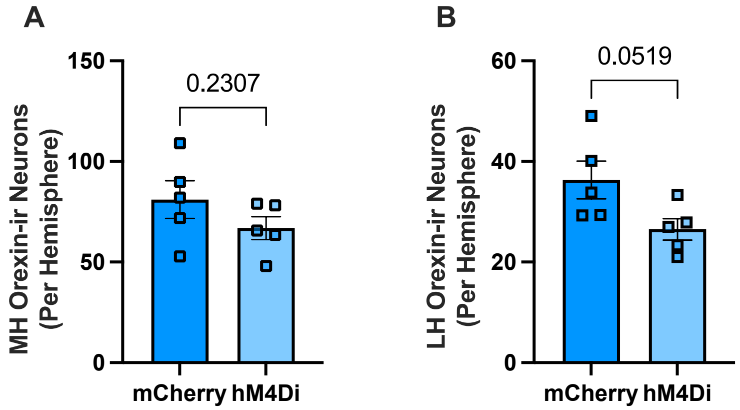

Supplemental Figure 2. Chronic inhibition of orexin neurons during oxycodone reveals fewer orexin cell numbers in LH. Rats injected with mCherry control virus (n=5) or hM4Di (n=5) were given 21d of twice daily oxycodone with CNO. On Day 22, tissue was collected for orexin immunohistochemistry. **A)** Numbers of MH orexin neurons were not significantly reduced in rats that received chronic hM4Di inhibition of orexin neurons. **B)** However, in LH, there was a decrease in orexin neuron numbers following chronic hM4Di inhibition of orexin neurons. Unpaired t-tests.

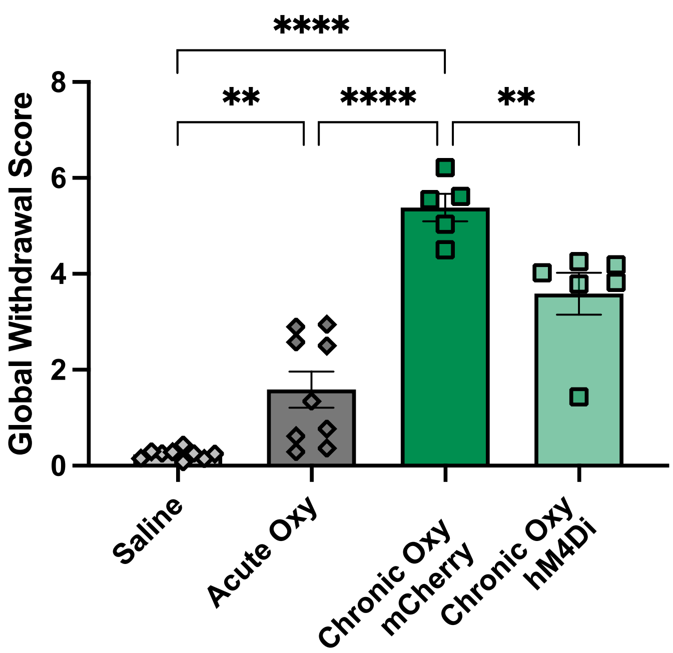

Supplemental Figure 3. Orexin neuron inhibition attenuates physical withdrawal. Following 21d of oxycodone treatment (no daily CNO), orexin-cre rats that had received either mCherry control (n=5) or hM4di (n=6) DIO-AAV were given CNO, followed by 30 min later with oxycodone, which was followed another 30 min later by naloxone. Additional groups of drug-naïve littermates (no viral transduction or oxycodone) received a single injection of saline (n=9) or oxycodone (n=9) 30 min following CNO, followed 30 min later by naloxone. A single oxycodone injection resulted in higher physical withdrawal than a single saline injection. Prior chronic oxycodone resulted in greater physical withdrawal than for acute oxycodone, indicating dependence seen with potentiated withdrawal. CNO + oxycodone before naloxone-precipitated withdrawal in hM4Di rats that had received chronic oxycodone attenuated physical withdrawal. One-way ANOVA with Holm-Sidak's tests, **p<0.01, ****p<0.0001.
